# Sequential Traumatic and Non-traumatic Slipped Upper Femoral Epiphysis in a Young Girl: A Case Report and Review of Diagnostic Challenges

**DOI:** 10.7759/cureus.92090

**Published:** 2025-09-11

**Authors:** Muhammad Amjad, Abdul Rehman

**Affiliations:** 1 Department of Emergency Medicine, St. Luke's General Hospital, Kilkenny, IRL

**Keywords:** atraumatic limp, paediatric groin pain, pediatric hip pain, slipped capital femoral epiphysis, slipped upper femoral epiphysis

## Abstract

Slipped upper femoral epiphysis (SUFE), also known as slipped capital femoral epiphysis, is a serious adolescent orthopedic condition, with potential for bilateral involvement. While most cases are idiopathic and occur during periods of rapid growth, trauma can unmask underlying physeal instability. We present a rare case of a young girl with a normal body mass index (BMI) who developed a traumatic SUFE in the left hip, followed one year later by a delayed, non-traumatic SUFE in the right hip. The delayed diagnosis of contralateral hip involvement underscores the diagnostic complexities of early or subtle SUFE and the importance of ongoing clinical vigilance, even in the presence of normal radiographs. This case supports a more proactive surveillance approach for the contralateral hip in pediatric SUFE patients.

## Introduction

Slipped upper femoral epiphysis (SUFE) is an adolescent hip disorder characterized by displacement through the proximal femoral physis. The epiphysis remains seated within the acetabulum, while the metaphysis undergoes external rotation and anterior translation. The slippage typically occurs at the hypertrophic zone of the physis. The condition most often presents between ages 10 and 16 years and is slightly more common in males, with a male: female ratio of approximately 3:2 [1]. Bilateral involvement occurs in up to 40% of cases [2].

Risk factors include obesity, male sex, African American ethnicity, endocrinopathies (e.g., hypothyroidism, growth hormone abnormalities), renal osteodystrophy, and radiation exposure [3]. While many cases are idiopathic, trauma can act as a precipitating factor in an already weakened physis. The diagnosis can be missed or delayed, especially in early or atypical presentations, or in the absence of clear radiographic findings [4].

We present the case of a young girl who initially developed traumatic SUFE of the left hip and, one year later, experienced delayed non-traumatic contralateral SUFE. This case underscores the importance of maintaining a high index of suspicion for bilateral involvement and revisiting current guidelines on contralateral surveillance and management.

## Case presentation

A previously healthy 11-year-old girl, with no significant past medical or surgical history and a normal BMI of 19.0 kg/m², presented to the injury clinic with a six-hour history of acute left hip pain following a fall while playing basketball at school. She reported difficulty bearing weight on the affected side. On physical examination, she exhibited a minimal limp and pain on internal rotation and abduction of the left hip, with restriction at the terminal range of motion. Flexion and external rotation were relatively preserved. Anterior and lateral hip tenderness was noted, without local signs of trauma, swelling, or infection. There was no local swelling and no signs of trauma or infection. Distal neurovascular examination of the left lower limb was normal.

**Figure 1:**
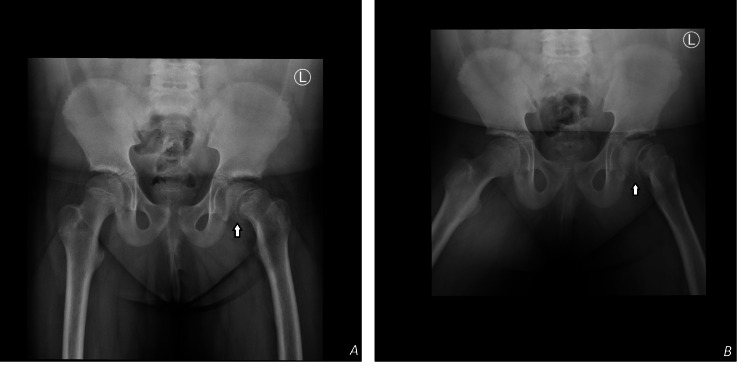
X-rays of the pelvis, AP (A) and frog-leg (B) views, showing SUFE of the left hip joint (white arrows). SUFE: Slipped Upper Femoral Epiphysis; AP: Anteroposterior

**Figure 2:**
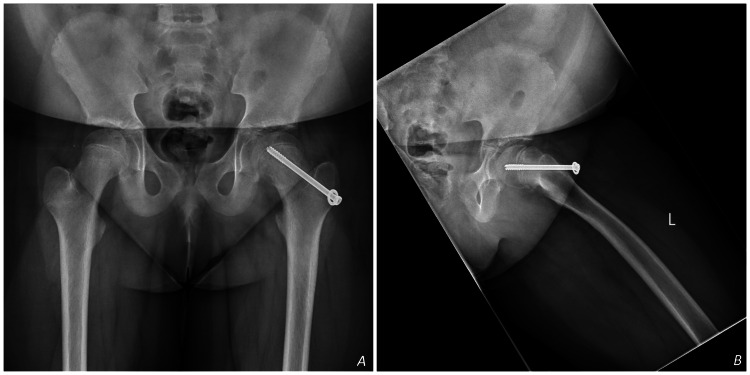
Post-operative X-rays, AP view of the pelvis (A) and Dunn view (B) of the left hip, showing percutaneous pin fixation of SUFE in the left hip. SUFE: Slipped Upper Femoral Epiphysis; AP: Anteroposterior

Pelvic radiography revealed SUFE in the left hip with partially closed triradiate cartilage (Figure 1). She was urgently referred to orthopedic services and underwent successful in-situ percutaneous pin fixation, using a single partially threaded cannulated cancellous screw and a washer (Figure 2). No capsular decompression was performed during the procedure, and a fracture table was used to maintain limb position and aid guidewire placement. The postoperative course was uneventful, with complete recovery of hip function.

**Figure 3:**
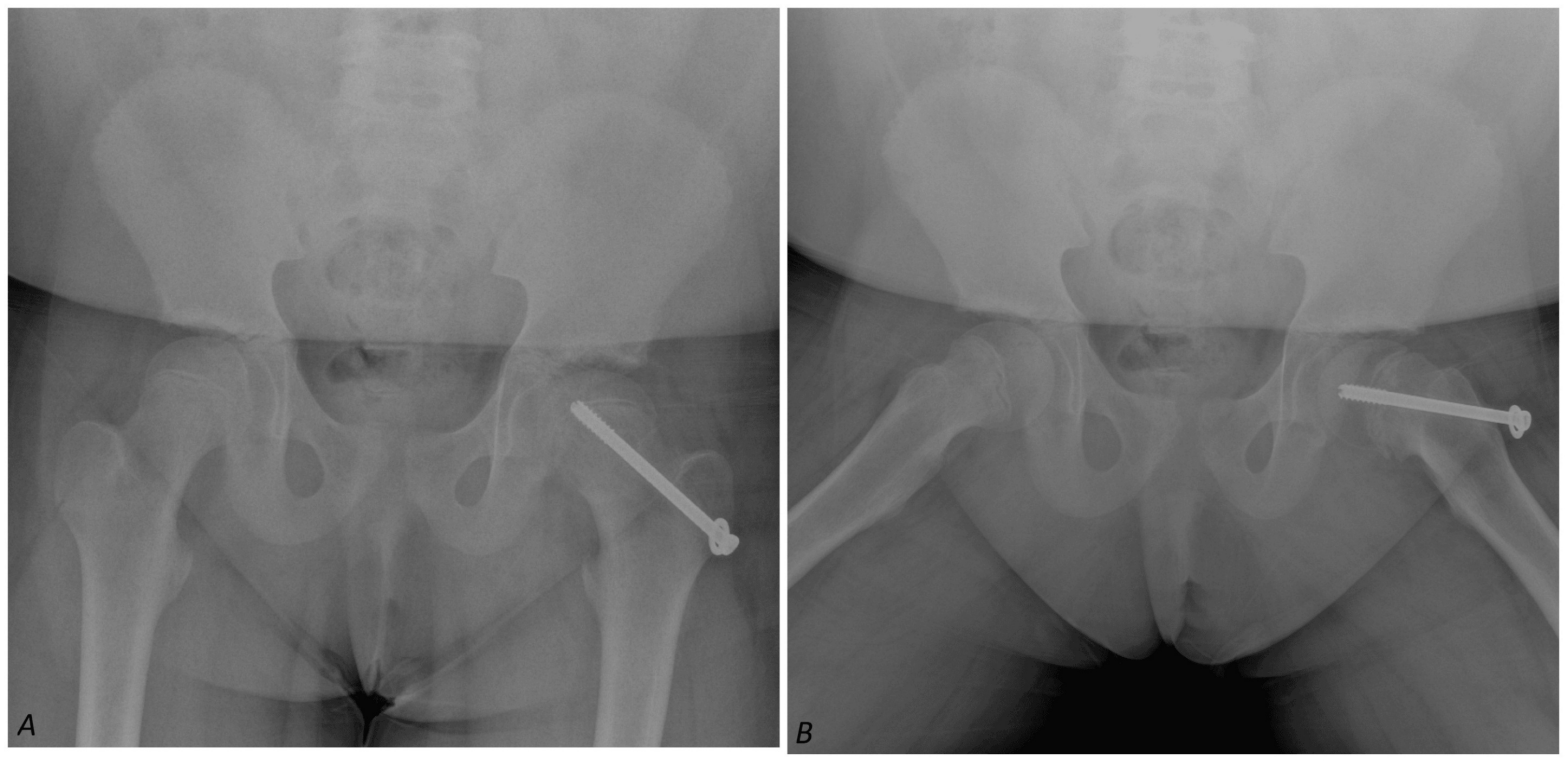
AP (A) and frog leg (B) X-ray views, showing pin fixation of SUFE in left hip without any evidence of SUFE in the right hip. SUFE: Slipped Upper Femoral Epiphysis, AP: Anteroposterior

One year later, the patient re-presented with a two-week history of intermittent, non-traumatic right groin pain. She was fully weight-bearing with no gait abnormality. Physical examination revealed a full range of motion in both hips, with no local or systemic signs of infection. Distal neurovascular examination of the right lower limb was normal. Repeat pelvic X-rays were unremarkable (Figure 3). She was discharged with conservative advice, including rest and physiotherapy.

**Figure 4:**
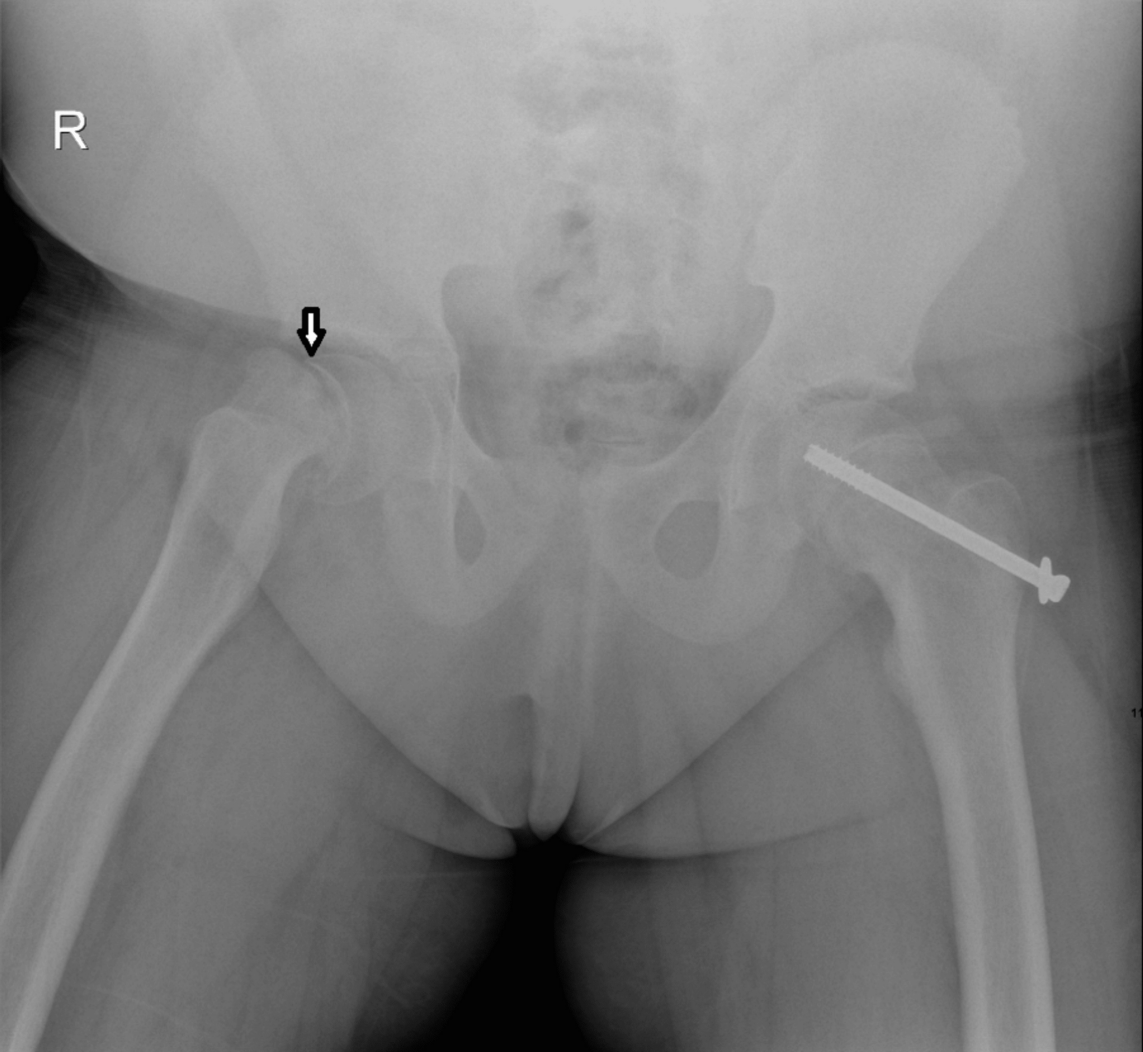
X-ray pelvis (AP view) showing SUFE (white arrow) in the right hip and previous percutaneous pin fixation in the left hip. SUFE: Slipped Upper Femoral Epiphysis; AP: Anteroposterior

**Figure 5:**
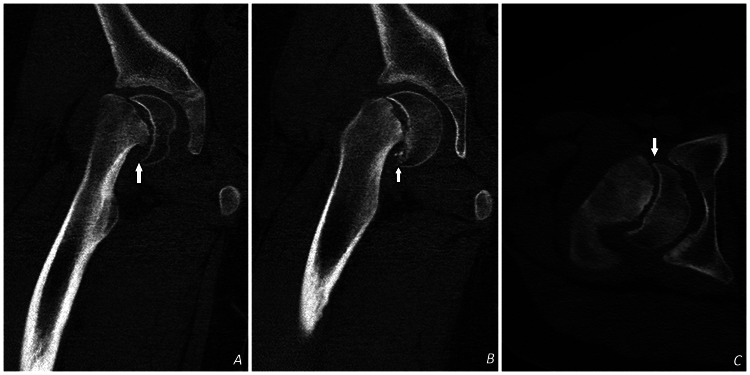
Axial (C) and coronal (A and B) CT scan views of the right hip demonstrating SUFE (white arrows). SUFE: Slipped Upper Femoral Epiphysis

**Figure 6:**
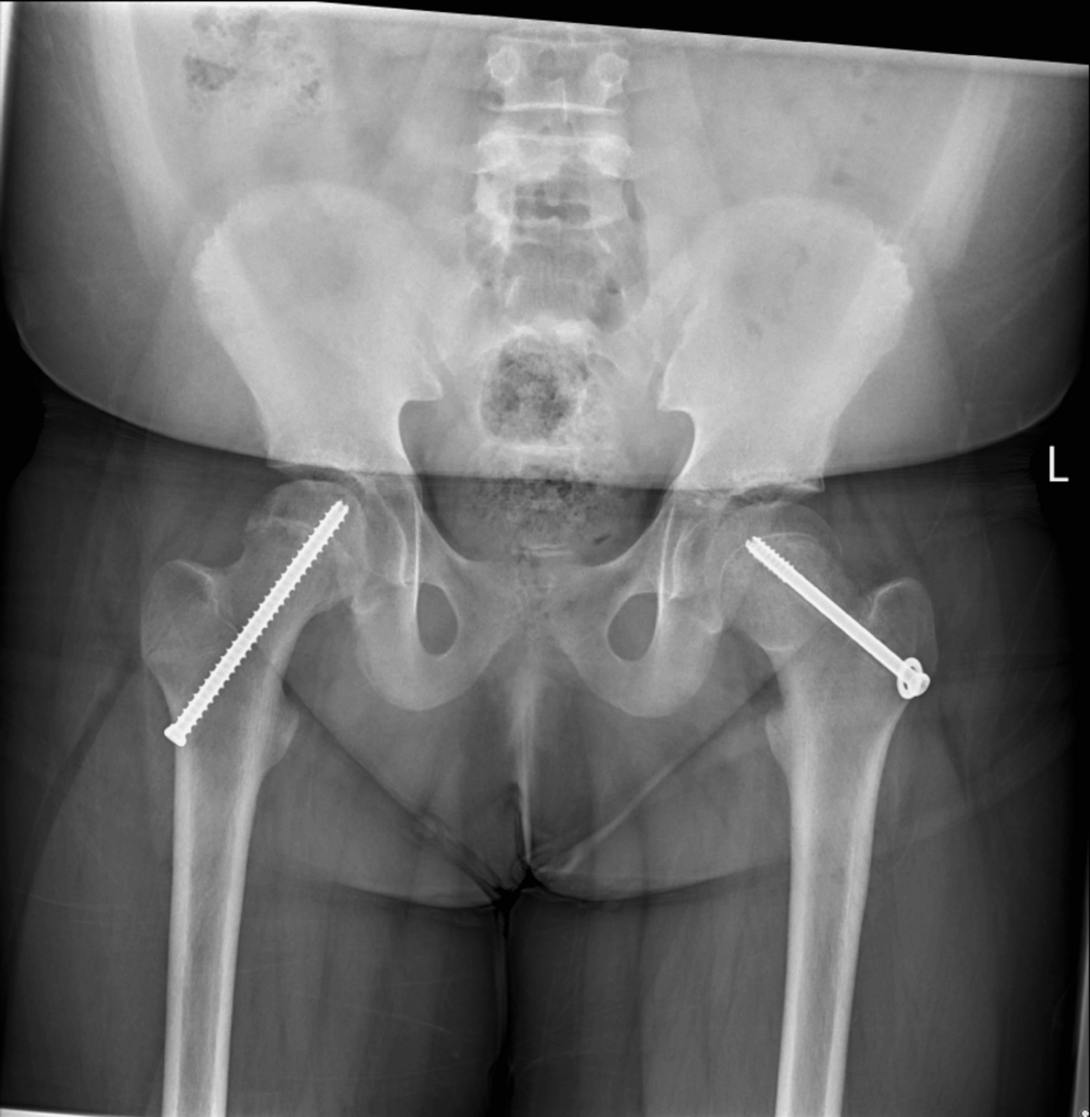
Post-operative X-ray pelvis (AP view) showing pin fixation in bilateral hips. AP: Anteroposterior

Three months later, she returned with worsening right hip pain and inability to bear weight. This time, repeat radiographs and a CT scan revealed a right-sided SUFE (Figures 4, 5). She was again referred to orthopedic surgery and underwent successful in-situ pin fixation of the right hip, using a single fully threaded cannulated cancellous screw (Figure 6). No deliberate attempt at reduction was made; a small degree of serendipitous reduction occurred during patient positioning, which was accepted and followed by in-situ pinning to minimize the risk of avascular necrosis (AVN). No capsular decompression was performed during the procedure, and a fracture table was used to maintain limb position and aid guidewire placement. Her recovery was uneventful.

## Discussion

SUFE is the most common hip disorder in adolescents, with incidence ranging from 0.33 to 24.6 per 100,000 children, depending on the geographic region and demographic variables [4]. The condition typically presents between 10 and 16 years of age but may occasionally occur earlier, especially in patients with underlying endocrinopathies or growth abnormalities [5]. Although our patient was 11 years old at the time of her initial presentation, the presence of a normal BMI and a preceding traumatic event rendered the presentation atypical for SUFE.

The exact etiology of SUFE remains multifactorial, with the physis considered the point of mechanical weakness during rapid growth. Known risk factors include: (i) Obesity: Increased mechanical stress across the physis [6]; (ii) Sex and Race: More common in males and African American children [7]; (iii) Endocrinopathies: Hypothyroidism, growth hormone deficiency, and panhypopituitarism [7]; (iv) Renal disorders and radiation therapy are also associated with physeal instability [7].

Bilateral SUFE occurs in 20-40% of cases. A study by Loder et al. showed that 80-90% of patients developed contralateral slips within 18 months [8]. The risk is higher in patients younger than 10 years, those with endocrinopathies, and in cases with initial severe slips. Our patient developed contralateral SUFE one year after the initial event, consistent with the known risk window.

In our case, no endocrine or systemic risk factors were identified. The initial trauma likely unmasked underlying physeal vulnerability. The contralateral hip, while asymptomatic and initially radiographically normal, eventually developed SUFE, highlighting that idiopathic or non-traumatic SUFE may follow an isolated traumatic presentation.

The mainstay of SUFE treatment is surgical stabilization. In situ pinning is most common for stable slips, whereas unstable slips may require open reduction and internal fixation [9]. In our case, both hips underwent successful pinning. Postoperative care includes restricted weight-bearing and close follow-up. Long-term surveillance is essential to monitor for any complications and contralateral involvement.

Routine monitoring of the contralateral hip in SUFE remains a subject of ongoing debate [10]. While prophylactic pinning of the asymptomatic side is not universally recommended, it may be considered in high-risk cases, especially in very young patients or when the initial slip is severe [2]. MRI is the most sensitive imaging modality for detecting early or pre-slip changes in the contralateral hip; however, it is not routinely performed due to cost and access issues [11].

The potential complications of SUFE are well recognized and can significantly affect long-term outcomes. AVN, particularly in unstable slips, is among the most serious sequelae [12]. Chondrolysis of the hip, characterized by progressive joint space narrowing due to cartilage loss, may also occur and result in severe functional limitation. Residual deformity of the proximal femur can lead to femoroacetabular impingement (FAI), further predisposing patients to hip dysfunction. Ultimately, untreated or inadequately managed cases may progress to early-onset hip osteoarthritis, highlighting the importance of timely diagnosis and appropriate management.

**Figure 7:**
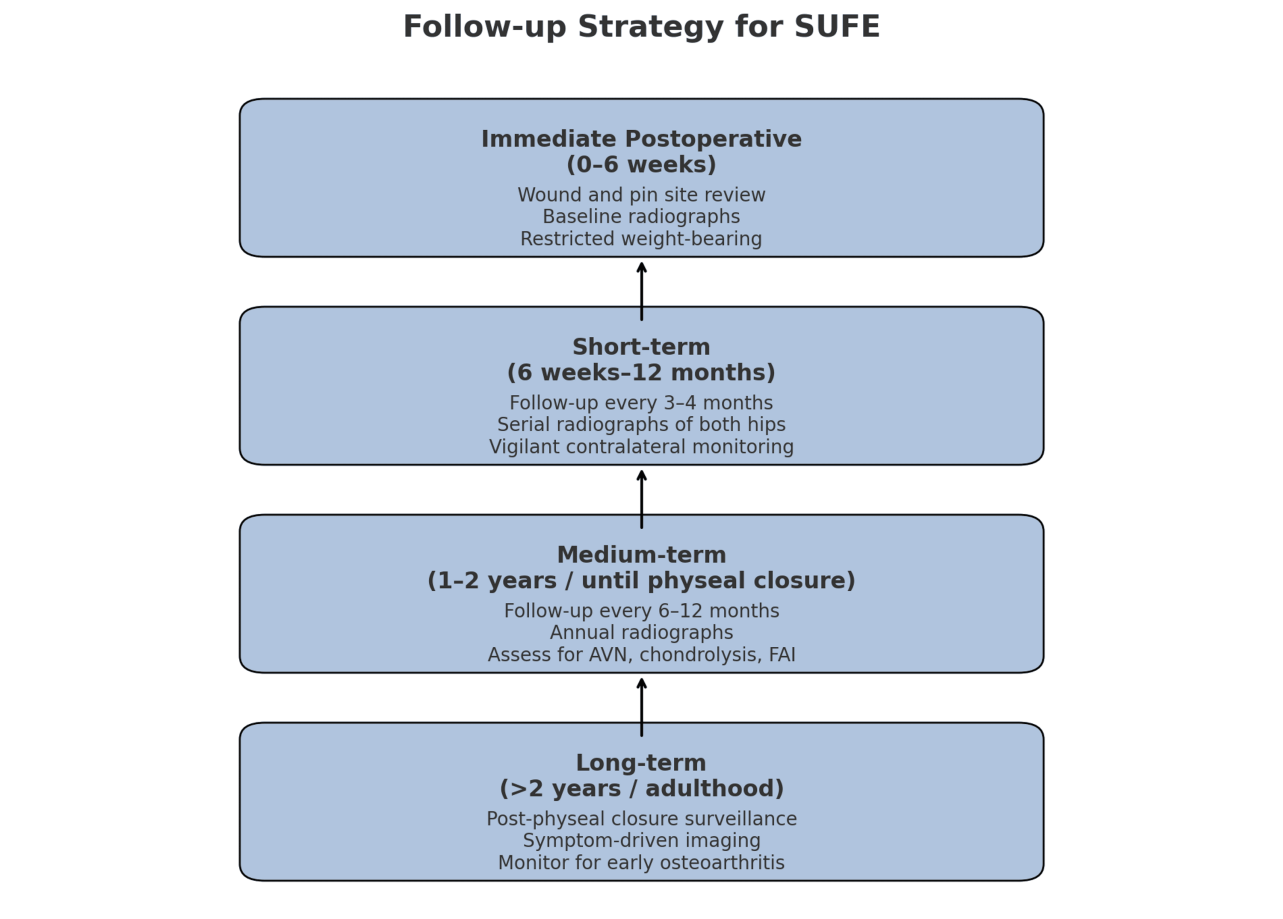
Suggested follow-up strategy for slipped upper femoral epiphysis (SUFE). The diagram outlines a structured approach to postoperative and long-term monitoring. In the immediate postoperative period (0–6 weeks), focus is placed on wound and pin site review, baseline radiographs, and restricted weight-bearing. The short-term phase (6 weeks–12 months) involves 3–4 monthly clinical reviews, serial radiographs, and careful monitoring of the contralateral hip, with consideration of prophylactic pinning or MRI surveillance in high-risk patients. The medium-term phase (1–2 years, until physeal closure) emphasizes annual radiographs, continued clinical reviews, and monitoring for complications such as avascular necrosis (AVN), chondrolysis, and femoroacetabular impingement (FAI). In the long-term phase (adolescence to adulthood), follow-up is intermittent and symptom-driven, with the goal of detecting early sequelae such as impingement and osteoarthritis.

Our suggested follow-up strategy for SUFE is structured across four phases (Figure 7). In the immediate postoperative period (0-6 weeks), emphasis should be placed on wound and pin site assessment, evaluation of pain and mobility, and obtaining baseline radiographs to confirm implant position. During this phase, patients should be advised to maintain restricted weight-bearing, typically with crutches and toe-touch or partial weight-bearing until clearance is given. In the short-term period (6 weeks-12 months), patients should undergo clinical reviews every 3-4 months to assess gait, hip range of motion, and leg length discrepancy. Radiological follow-up with anteroposterior pelvis and frog-leg lateral views should be considered at similar intervals throughout the first year. Particular attention should be paid to the contralateral hip, with careful monitoring for symptoms such as groin or hip pain, or knee pain and limping. In high-risk patients, younger than 10 years, with endocrinopathies, or with an initial severe slip, prophylactic pinning or MRI surveillance may be considered. In the medium-term phase (1-2 years or until physeal closure), follow-up should occur every 6-12 months, with annual radiographs or earlier if symptoms arise. Clinical and imaging surveillance should also focus on detecting complications such as AVN, chondrolysis, and FAI. Finally, in the long-term phase (adolescence to adulthood), intermittent follow-up after physeal closure is advised, guided primarily by symptoms, with imaging performed as clinically indicated. The principal objective during this period is the early detection of sequelae, including impingement and the development of osteoarthritis.

Fortunately, our patient did not experience any complications from the delayed diagnosis and had an uneventful postoperative course; however, the delay in diagnosing and the lack of consideration for prophylactic fixation of the contralateral SUFE represent a missed opportunity for earlier intervention.

## Conclusions

This case highlights the diagnostic complexity and clinical progression of SUFE in a child who initially presented with a traumatic unilateral slip, followed by a delayed, non-traumatic contralateral slip. The presence of normal interim imaging and the absence of typical clinical symptoms may lead to false reassurance and delayed diagnosis. Given the well-documented risk of bilateral involvement, particularly within the critical two-year window following the initial presentation, clinicians must maintain a high index of suspicion for contralateral SUFE, even in asymptomatic patients and in the absence of trauma or radiographic abnormalities. Regular clinical follow-up, a low threshold for repeat and higher imaging, and individualized risk stratification for prophylactic fixation should be considered to prevent diagnostic delays and reduce the risk of long-term complications.
